# Prognostic factors of *Pneumocystis* pneumonia in patients with systemic autoimmune diseases

**DOI:** 10.1371/journal.pone.0214324

**Published:** 2019-03-25

**Authors:** Takahiro Kageyama, Shunsuke Furuta, Kei Ikeda, Shin-ichiro Kagami, Daisuke Kashiwakuma, Takao Sugiyama, Takeshi Umibe, Norihiko Watanabe, Mieko Yamagata, Hiroshi Nakajima

**Affiliations:** 1 Department of Allergy and Clinical Immunology, Chiba University Hospital, Chiba, Japan; 2 Research Center for Allergy and Clinical Immunology, Asahi General Hospital, Chiba, Japan; 3 Department of Rheumatology, National Hospital Organization Shimoshizu Hospital, Chiba, Japan; 4 Rheumatology Center, Matsudo City General Hospital, Chiba, Japan; 5 Center for Rheumatic Diseases, Saiseikai Narashino Hospital, Chiba, Japan; Keio University, JAPAN

## Abstract

**Objective:**

*Pneumocystis* pneumonia (PCP) is one of the most common opportunistic infections. In systemic autoimmune disease patients receiving immunosuppressive treatments, low lymphocyte count, old age and coexisting lung disease have been known as risk factors for the occurrence of PCP. However, factors relevant to prognosis of PCP have not been fully studied.

**Methods:**

A total of 95 sequential patients who developed PCP during immunosuppressive treatment for systemic autoimmune diseases was identified from five Japanese centres. We retrospectively assessed baseline characteristics, immunosuppressive treatment prior to the onset of PCP, treatment for PCP and survival. Univariate and multivariate analyses were performed to identify prognostic factors.

**Results:**

Forty-two deaths (44.2%) were observed in this study. Age at the diagnosis of PCP was higher in non-survivors than in survivors (74 years vs. 64 years, p = 0.008). Non-survivors more frequently had lung involvement than did survivors (47.6% vs. 13.2%, p<0.001). Median lymphocyte count at the diagnosis of PCP was lower in non-survivors than in survivors (499/μl vs. 874/μl, p = 0.002). Multivariate analysis identified lower lymphocyte count, older age and coexisting lung disease at the diagnosis of PCP as independent risk factors for death. Those risk factors for death were similar to the known risk factors for the occurrence of PCP.

**Conclusion:**

Although PCP can occur even in patients without these risk factors, our data demonstrate that the overall prognosis of PCP in such patients is good. Given that the standard prophylactic treatment against PCP has safety issues, the risk-stratified use of prophylactic treatment may be advisable.

## Introduction

*Pneumocystis* pneumonia (PCP) is a pneumonia caused by a yeast-like fungus *Pneumocystis jirovecii*. PCP is widely recognized as an opportunistic infection and usually occurs in immunocompromised patients due to human immunodeficiency virus (HIV) infection, chemotherapy for malignancy, and immunosuppressive treatment for systemic autoimmune diseases and organ transplantation.

PCP can develop respiratory failure and be a life-threatening condition [1]. In patients with systemic autoimmune diseases, it has been reported that a higher dose of glucocorticoids is associated with higher risk for occurrence of PCP [2]. Thus, prophylaxis for PCP using trimethoprim/sulfamethoxazole (TMP/SMX) is now widely used when patients with autoimmune diseases receive a moderate to high dose of glucocorticoids [3]. The prophylaxis has dramatically reduced the occurrence of PCP in such patients. On the other hand, it has recently been reported PCP occasionally occurs in patients receiving low-dose glucocorticoids or receiving only disease modifying anti-rheumatic drugs (DMARDs) and/or biologic agents [4]. However, it remains unclear whether the indication of prophylaxis for PCP should be extended to such patients or not. This is a big issue especially for rheumatoid arthritis (RA) patients because most of RA patients do not require a moderate to high dose of glucocorticoids and require long-term DMARD and/or biologic treatment. If the prophylaxis is recommended for such patients, a large number of RA patients will need long-term TMP/SMX administration. Although the effect of TMP/SMX prophylaxis on preventing PCP has been established, long-term TMP/SMX administration potentially causes various side effects such as cytopenia, toxicoderma, liver dysfunction and electrolyte abnormality [5].

Considering the indication of prophylaxis, not only the incidence of the disease but also the prognosis of the disease is quite important. Previous studies have identified high-dose glucocorticoids, lymphopenia, age and lung involvement as risk factors for the development of PCP [6–11]. Regarding mortality, Chen et al. identified low PaO2/FiO2 and Kim et al. identified high D(A-a)O2 and preexisting lung disease as independent risk factors for death [12,13]. However, risk factors for mortality related to the intensity and/or the class of immunosuppressive treatment prior to the development of PCP have not been identified. This is of particular interest because the influence of immune-suppression on the severity of PCP in autoimmune diseases can be either protective or detrimental. The excess host immune response to fungus bodies has been considered to exacerbate lung inflammation [14] and short-term high-dose glucocorticoid is often used in combination with antibiotics against PCP for this reason.

In this retrospective observational study, we aimed to identify risk factors for poor prognosis of PCP in systemic autoimmune disease patients, with a particular focus on the intensity and the class of immunosuppressive treatment they are receiving prior to the development of PCP.

## Patients and methods

### Patients

We retrospectively collected patients who developed PCP during immunosuppressive treatments for systemic autoimmune diseases from 5 Japanese centres: Chiba University Hospital, Asahi General Hospital, Saiseikai Narashino Hospital, National Hospital Organization Shimoshizu Hospital and Matsudo City General Hospital. Sequential 95 PCP patients, diagnosed between 2000 and 2015, were identified.

### Ethics

This study was approved by the ethics committee of Chiba University School of Medicine (reference number: 2239). The ethics committee waived the requirement for patient’s written informed consent because it was not necessary for local regulations in case of a retrospective observational study. All data fully anonymized before analyzing them. The data were shared in accordance with the plan approved by the ethics committee, and only the data used in this specific study were provided.

### Diagnosis

According to previous studies [15,16], the diagnosis of PCP was considered as definitive if *P*. *jirovecii* was found on the microscopical analysis of respiratory samples from patients with clinical manifestations (pyrexia, dry cough, or dyspnea), hypoxemia, and radiologic findings compatible with PCP. The diagnosis of PCP was considered as presumptive if patients met all three criteria and had either a positive polymerase-chain-reaction (PCR) test for *P*. *jirovecii* DNA or an increased serum level of β-D-glucan with an appropriate response to the standard treatments for PCP. Both definitive and presumptive cases were included in this study.

### Assessment

We retrospectively assessed age at diagnosis of PCP, sex, diagnosis of autoimmune diseases, presence/absence of lung involvement prior to PCP, treatments for the autoimmune diseases including the dose of glucocorticoids, the use of immunosuppressants and the use of biologics, serum β-D-glucan, LDH, CRP and KL-6 levels at diagnosis of PCP. Information on treatment for PCP and mortality was also collated. In this study, “death” was defined as death that occurred during the treatment period for PCP. Data were acquired from patients’ medical charts and computer records.

### Statistics

The distributions were described by median and interquartile range, and compared by Mann-Whitney U test. Proportions were compared by chi-square test, or Fisher’s exact test when the expected frequency was less than 5 in one or more cells. Risk factors for death were assessed by univariate and multivariate analysis with a logistic regression model. All analyses were performed by using IBM SPSS Statistics version 23 and p*<0*.*05* was taken to indicate statistical significance.

## Results

### Survivor vs. non-survivor

We evaluated a total of 95 patients with PCP which occurred during immunosuppressive treatment for systemic autoimmune diseases. Three patients were microscopic analysis-positive and diagnosed as definitive PCP. In patients diagnosed as presumptive PCP, serum β-D-glucan levels at the onset were elevated in 85 of 92 patients, while 37 of 43 patients who obtained respiratory samples had positive PCR test for *P*. *jirovecii* DNA. Forty-five patients had RA and 50 patients had non-RA autoimmune diseases, which included vasculitis syndrome (n = 20), polymyositis/dermatomyositis (n = 11), systemic lupus erythematosus (n = 7), systemic sclerosis (n = 3), mixed connective tissue diseases (n = 2), Sjogren’s syndrome (n = 2), relapsing polychondritis (n = 1), IgG4-related disease (n = 1), polymyalgia rheumatica (n = 1), anti-phosholipid syndrome (n = 1), idiopathic interstitial pneumonia (n = 1), and pemphigoid (n = 1). Forty-two deaths (44.2%) were observed in this study.

Baseline characteristics, immunosuppressive treatments for autoimmune diseases at diagnosis of PCP, blood examinations at diagnosis of PCP and treatments for PCP were compared between survivors and non-survivors (Table 1). Age at diagnosis of PCP was significantly higher in non-survivors than survivors (74y vs. 64y, p = 0.008). Non-survivors more frequently had lung involvement prior to PCP than did survivors (47.6% vs. 13.2%, p<0.001).

**Table 1:** Comparison between survivors vs. non-survivors in PCP patients.

	Overall (n = 95)	Survivor (n = 53)	Non-Survivor (n = 42)	*P* values
**Baseline characteristics**				
Age, year (IQR)	69 (61–78)	64 (59.5–75)	74 (64–79)	.008
Male: female (female rate)	38: 57 (60.0)	22: 31 (58.4)	16: 26 (61.9)	.736
Diagnosis, RA: non-RA	45: 50	31: 22	14: 28	.015
Lung involvement n (%)	27 (28.4)	7 (13.2)	20 (47.6)	< .001
**Treatment for autoimmune diseases at diagnosis of PCP**				
Glucocorticoids-use n (%)	87 (91.5)	46 (86.7)	41 (97.6)	.073
Dose^≠^ mg/day (IQR)	15 (8–25)	12.5 (5.75–20)	20 (10–30)	.006
High-dose use^≠^ n (%)	9 (9.5)	1 (1.8)	8 (19.0)	.009
IS-use n (%)	60 (63.1)	34 (64.1)	26 (61.9)	.822
Cyclophosphamide n (%)	13 (13.7)	4 (7.5)	9 (21.4)	.051
Cyclosporine A n (%)	4 (4.2)	2 (3.8)	2 (4.6)	>.999
Tacrolimus n (%)	9 (9.5)	6 (11.3)	3 (7.1)	.727
Methotrexate n (%)	45 (47.4)	32 (60.4)	13 (30.9)	.004
Biologics-use n (%)	16 (16.8)	12 (22.6)	4 (9.5)	.090
Prophylaxis for PCP n (%)	5 (5.2)	2 (3.7)	3 (7.1)	.652
**Blood examination at diagnosis of PCP**				
Lymphocyte count /μl (IQR)	744.0 (318.0–1027.0)	874.2 (540.5–1265.5)	499.75 (244.0–795.4)	.002
β-D-Glucan pg/ml (IQR)	81.4 (36.4–263.0)	67.6 (25.1–263.6)	114.6 (51.4–250.2)	.111
LDH U/l (IQR)	388 (297–539)	333 (261–434)	489 (385.5–626.25)	< .001
KL-6 U/ml (IQR)	744.5 (439.5–1107.5)	651 (346.5–932)	875 (669–1590)	.002
CRP mg/l (IQR)	7.2 (2.8–11.4)	5.3 (2.0–9.5)	9.7 (3.4–18.1)	.016
**Treatment for PCP**				
TMP/SMX n (%)	92 (96.8)	50 (94.3)	42 (100)	.252
Atovaquone n (%)	16 (16.8)	12 (22.6)	4 (9.5)	.090
Pentamidine n (%)	26 (27.3)	10 (18.8)	16 (38.0)	.037
Initial dose of glucocorticoids^≠^ mg/day (IQR)	40 (21.25–50)	40 (20–40)	42.5 (40–60)	.002

IQR: interquartile range; PCP: *Pneumocystis jiroveci* pneumonia; RA: rheumatoid arthritis; pred.: prednisolone; IS: immunosuppressants; CRP: C-reactive Protein; TMP/SMX: trimethoprim/sulfamethoxazole

^≠^Dose was converted to equivalent prednisolone dose. High-dose was defined as = <40mg/day.

The distributions of age, glucocorticoids dose, β-D-Glucan, KL-6, LDH and CRP were shown as median values with interquartile ranges.

The majority of patients in both groups received glucocorticoids for their diseases prior to the onset of PCP, and the median daily dose of prednisolone at the diagnosis of PCP was significantly higher in non-survivors than in survivors (20 mg vs. 12.5 mg, p = 0.006). Sixty patients (63.1%) received immunosuppressants. Cyclophosphamide-use showed tendency of higher mortality rate, while methotrexate-use was associated with lower mortality rate. Majority of MTX-users were RA patients (40/45) and treated with no or low-dose glucocorticoids Majority of CPA-users were vasculitis patients (10/13) and treated with high-dose glucocorticoids. Five patients (two survivors and three non-survivors) were receiving TMP/SMX prophylaxis at the diagnosis of PCP. In all centres participating this study, TMP/SMX prophylaxis is usually given during high-dose glucocorticoids treatment if patients do not have contraindication to TMP/SMX or unacceptable side effects of TMP/SMX.

Lymphocyte counts were significantly lower in non-survivors than survivors (p = 0.002). Serum LDH, KL-6 and CRP levels were significantly higher in non-survivors than in survivors (p<0.001, p = 0.002, P = 0.016).

After the diagnosis of PCP, all patients were initially treated with TMP/SMX except for three cases with pentamidine. Fifty-eight patients continued to receive TMP/SMX. Thirty-four of 92 patients had to discontinue TMP/SMX due to its side effects, such as rash, fever, liver dysfunction, electrolyte abnormality, renal disorder and cytopenia. Twelve patients switched from TMP/SMX to atovaquone, while 22 patients to pentamidine. Three out of these 22 patients finally received atovaquone due to side effects of pentamidine. In addition to treatment with antibiotics, glucocorticoids were used for preventing excessive host immune response to fungus bodies. Initial doses of glucocorticoids were significantly higher in non-survivors than survivors (p = 0.002).

### Univariate and multivariate analyses for death

All items of baseline characteristics, treatment for autoimmune disease at diagnosis of PCP, and lymphocyte count in Table 1 were selected as potential explanatory variables. We confirmed in advance there was no collinearity between them. Explanatory variables with p values less than 0.10 in univariate analysis were entered in the multivariate logistic regression model. Older age, the presence of lung involvement and lower lymphocyte count at diagnosis of PCP were identified as independent risk factors for death by multivariate analysis (Table 2). No death was observed in the patients without those identified risk factors.

**Table 2:** Univariate and multivariate analyses for death.

	Univariate			Multivariate		
	OR	95%CI	P values	OR	95%CI	P values
Age year						
<65	reference			reference		
65≤	3.32	1.36–8.10	0.008	8.68	2.26–33.48	0.002
Diagnosis						
Non-RA	reference			reference		
RA	0.35	0.15–0.82	0.016	0.62	0.14–2.80	0.538
Presence of lung involvement	5.97	2.19–16.23	<0.001	4.35	1.36–13.91	0.013
Lymphocyte count /μl						
1500≤	reference			reference		
500–1500	7.33	0.87–61.33	0.066	5.43	0.53–56.19	0.155
<500	19.25	2.20–168.00	0.007	29.92	2.42–370.68	0.008
Dose of daily prednisolone (per 10mg increase)	1.66	1.16–2.37	0.005	1.47	0.82–2.66	0.194
Use of biologics	0.36	0.11–1.21	0.099	0.86	0.16–4.84	0.873

RA: rheumatoid arthritis; OR: odds ratio; CI: confidence interval

### Mortality according to lymphocyte count, age and a dose of glucocorticoids at diagnosis of PCP

We calculated mortality rate according to lymphocyte counts at diagnosis of PCP (Table 3(A)). One of 11 patients (9.0%) died from PCP in the normal range group (≥1500/μl), while two-thirds of the patients died in the severe lymphopenia group (<500/μl). One death in the normal range group was a systemic sclerosis patient with lung involvement whose age was in the seventies, and was treated with 10mg/day prednisolone monotherapy. Her lymphocyte count at the diagnosis of PCP was 1704/μl.

**Table 3:** Mortality rates according to lymphocyte count, age and a dose of glucocorticoids at diagnosis of PCP.

	Number of patients	Number of death (%)
Lymphocyte count /μl		
<250	18	11 (61.1)
250–500	15	10 (66.6)
500–1000	37	13 (35.1)
1000–1500	14	7 (50.0)
1500 = <	11	1 (9.0)
Age year		
<50	7	2 (28.5)
51–65	35	9 (25.7)
66–80	44	27 (61.3)
81 = <	9	4 (44.4)
Daily dose of prednisolone mg		
<5	15	2 (13.3)
5–10	17	6 (35.2)
10–20	26	13 (50.0)
20–30	17	9 (52.9)
30 = <	20	12 (60.0)

We also calculated mortality rates according to age groups (Table 3(B)). When the patients were younger than 65 years old, the mortality rate of PCP was 26.1%. On the other hand, the mortality rate increased to 58.4% in the patients aged 65 years or older.

Although the dose of glucocorticoids for autoimmune diseases at the onset of PCP was significantly associated with mortality only in the univariate analysis but not in the multivariate analysis, we also calculated mortality rates according to the dose groups of glucocorticoids as we planned. As shown in Table 3(C), dose-dependent elevation of mortality rates was observed. Of the two cases in the lowest dose group (<5mg/day prednisolone), one was a RA patient whose age was in the eighties, and was treated with 2.5mg/day of prednisolone, 6mg/week of methotrexate and 0.5mg/day of tacrolimus. The other was also a RA patient whose age was in the eighties, and was treated with only 10mg/week of methotrexate. Their lymphocyte counts at the diagnosis of PCP were 285/μl and 192/μl, respectively.

## Discussion

This study aimed to investigate prognostic factors of PCP which occurred during immunosuppressive treatment for systemic autoimmune diseases. Multivariate analysis identified lower lymphocyte count, coexisting lung involvement and older age at the diagnosis of PCP as risk factors for death. These risk factors for death were the same as known risk factors for the occurrence of PCP [7]. While PCP can occur even in patients without those risk factors as previously reported, our data demonstrate that the overall prognosis of PCP in such patients is good. Given that the standard prophylactic treatment against PCP has safety issues, the risk-stratified use of prophylactic treatment may be advisable. Autoimmune disease patients without those risk factors may not need prophylactic treatment for PCP.

Patients in this study were receiving various intensity and types of immunosuppressive treatment for underlying autoimmune diseases. However, none of the parameters related to prior immunosuppressive treatment was identified as a risk factor for death by multivariate analysis. Lymphocyte count, on the other hand, was identified as an independent risk factor for death in our study. We assume that lymphocyte count represents an overall effect of immunosuppressive treatment and can be a good biomarker. Because the low lymphocyte count is associated with poor prognosis, deep immunosuppression at the diagnosis of PCP does not seem to have a beneficial effect on overall survival.

In this study, serum LDH, KL-6 and CRP levels at diagnosis of PCP were significantly higher in the non-survivors than the survivors. High titers of those markers might just reflect more severe disease. However, treatment options for PCP were limited at the moment. We cannot select specific treatments for PCP according to severity of PCP if we can assess it using those markers.

Benefit of adjunctive glucocorticoids for PCP has been established in HIV patients [17]. It has been frequently used in non-HIV patients as well. However, retrospective studies reported conflicting results in non-HIV patients [14,18,19], and there are no standardised regimens. In this study initial dose and tapering schedule of adjunctive glucocorticoids were decided by each physician according to severity of PCP and baseline dose at the onset of PCP. The initial dose was significantly higher in non-survivors than survivors, probably because non-survivors were assessed as having more severe disease than survivors. The doses at other time points have not been evaluated in this study, though they might affect the survival. Future studies focused on this issue will be needed.

Low frequency of definitive PCP was observed in this study as well as previous studies with non-HIV patients [3,7,11]. Microscopical analysis of respiratory samples, ideally bronchoalveolar lavage fluid (BALF), is required for diagnosing definitive PCP. However, it is often difficult to obtain BALF because PCP in non-HIV patients present severe and rapidly progressive pneumonia [20]. In addition, it is known non-HIV patients often develop PCP with a lower parasite burden than HIV patients [21].

There were some limitations in this study. We have not assessed the length of treatment period and the cumulative dose of glucocorticoids prior to the onset of PCP, which might have influenced the prognosis. Moreover, difference among centres in a range of available supportive care for severe conditions such as extracorporeal membrane oxygenation might have influenced the mortality rate of PCP. However, the multicentre design in this study reduced the bias.

In conclusion, lower lymphocyte count, coexisting lung involvement and older age at diagnosis of PCP were identified as risk factors for death in patients who developed PCP during immunosuppressive treatment for systemic autoimmune diseases. Those risk factors for death were similar to the known risk factors for the occurrence of PCP. Combining our results and previous studies, the indication of prophylaxis for PCP in autoimmune diseases should be stratified by these risk factors. Continuous monitoring of lymphocyte count is important to appreciate an increased risk for PCP in individual patients receiving a various range of immunosuppressive treatment.

## Supporting information

S1 File Dataset. Individual data of each participant are shown.
(XLSX)
